# Oxysterol/chitotriosidase based selective screening for Niemann-Pick type C in infantile cholestasis syndrome patients

**DOI:** 10.1186/s12881-019-0857-0

**Published:** 2019-07-11

**Authors:** Anna V. Degtyareva, Tatiana Y. Proshlyakova, Marina S. Gautier, Dmitry N. Degtyarev, Elena A. Kamenets, Galina V. Baydakova, Denis V. Rebrikov, Ekaterina Y. Zakharova

**Affiliations:** 1 Kulakov National Medical Research Center for Obstetrics, Gynecology and Perinatology, Moscow, Russia; 2 Sechenov First Moscow State Medical University, Moscow, Russia; 3 Research Centre for Medical Genetics, Moscow, Russia; 4 Pirogov Russian National Research Medical University, Moscow, Russia

**Keywords:** Niemann-pick disease type C, Cholestasis, Biomarker, Oxysterol, Chitotriosidase, Screening*, NPC1, NPC2, JAG1, ABCB11, LARS*

## Abstract

**Background:**

Niemann-Pick disease type C (NP-C) is an inherited neurodegenerative disease (1 per 100 000 newborns) caused by NPC proteins impairment that leads to unesterified cholesterol accumulation in late endosomal/lysosomal compartments. To date the NP-C diagnostics is usually based on cholesterol detection in fibroblasts using an invasive and time-consuming Filipin staining and we need more arguments to widely introduce oxysterols as a biomarkers in NP-C.

**Methods:**

Insofar as NP-C represents about 8% of all infant cholestases, in this prospective observational study we tried to re-assess the specificity plasma oxysterol and chitotriosidase as a biochemical screening markers of NP-C in children with cholestasis syndrome of unknown origin. For 108 patients (aged from 2 weeks to 7 years) the levels of cholestane-3β,5α,6β-triol (C-triol) and chitotriosidase (ChT) were measured. For patients with elevated C-triol and/or ChT the *NPC1* and *NPC2* genes were Sanger-sequenced and 47 additional genes (from the custom liver damage panel) were NGS-sequenced.

**Results:**

Increased C-triol level (> 50 ng/ml) was detected in 4 (of 108) infants with cholestasis syndrome of unknown origin, with following molecular genetic NP-C diagnosis for one patient. Plasma cholesterol significantly correlates with C-triol (*p* < 0.05). NGS of high C-triol infants identified three patients with mutations in *JAG1* (Alagille syndrome) and *ABCB11* (Byler disease) genes. Increased ChT activity was detected in 8 (of 108) patients with various aetiologies, including NP-C, Byler disease and biliary atresia.

**Conclusion:**

Combined analysis of ChT activity and C-triol levels is an effective method for identifying NP-C.

## Background

Niemann-Pick disease type C (NP-C) is a rare progressive neurodegenerative disease with an incidence of one per 89 000–150 000 live births among the Western European population [1, 2]. Approximately 95% of NP-C cases are caused by mutations in the *NPC1* gene (locus 18q11-q12), with approximately 5% caused by mutations in the *NPC2* gene (locus 14q24.3). Pathological mutations in these genes lead to impaired intracellular cholesterol transport and subsequent accumulation of free cholesterol and lipids in endosomes and lysosomes [2].

The clinical symptoms of NP-C are highly variable and can be divided into three categories: visceral, neurological, and psychiatric [3]. Manifestations are variable in terms of the age ranging from the neonatal period (45–65%) up to the seventh decade of life [4–6].

The earliest clinical symptoms of the NP-C among all the patients are neonatal cholestasis syndrome, isolated splenomegaly and hepatosplenomegaly [1, 3, 7]. Some cases of nonimmune hydrops and ascites have also been reported [8, 9]. In most cases, signs of NP-C-related cholestasis reduce spontaneously by the 6–8th month of life, while liver and/or spleen enlargement persist for a long time [10].

Technically difficult and time-consuming Filipin staining was the basic diagnostic method in previous decades [11]. Since the modern NGS methods has not been considered as a screening approach yet (particularly in respect of high incidence of neonatal cholestasis with 1 per 2500 newborns) [3, 11, 12], the new biochemical markers are highly demanded for NP-C screening.

Chitotriosidase (ChT) is the macrophage activation associated plasma-borne enzyme that has been used as a biomarker for a lysosomal storage diseases (LSDs), including Gaucher disease (GD) and NP-C [13]. However, the NP-C specificity of this marker is not enough, being positive in various conditions including cholestasis and systemic autoimmune inflammatory diseases. Also ChT is not informative in a significant proportion of individuals because of a frequent loss-of-function variant, resulting in deficient activity in homozygous individuals, and possible false negative in heterozygous cases. Recently the reactive oxidative species (ROS), cholestan-3β,5α,6β-triol (C-triol) and 7-ketocholesterol (7-KC), have been established as reliable and convenient diagnostic biomarkers for NP-C [14–21]. Data (adult patients) indicates that C-triol has a good sensitivity and specificity for NP-C screening, while 7-KC has a limited specificity [11, 22].

Latest oxysterol profiling studies demonstrate a relatively low specificity of oxysterols as an NP-C biomarker in infants with cholestasis syndrome [23]. Insofar as NP-C represents about 8% of all infant cholestases [24], we tried to re-assess the specificity of C-triol and ChT plasma detection among infants with cholestasis syndrome for NP-C screening.

## Methods

### Ethics and consent

This prospective observational cohort study was carried out according to the Code of Ethics of the World Medical Association (Declaration of Helsinki) and the study protocol was reviewed and approved by the Ethics Committee of the Kulakov National Medical Research Center for Obstetrics, Gynecology and Perinatology (Protocol No.13 from Dec 06 2013). All participants (children’s parents) provided written informed consent.

### Patients and study design

The blood samples were obtained from 108 infants (aged from 2 weeks to 7 years) with cholestasis syndrome of unknown origin (documented during the first months of life) in Kulakov National Medical Research Center for Obstetrics, Gynecology and Perinatology (Moscow, Russia) and Research Centre for Medical Genetics (Moscow, Russia) between January 2014 and May 2017. The blood samples were collected in 4 ml BD Vacutainer K3EDTA tubes, centrifuged for 5 min at 3000 rpm and plasma was frozen at − 80 °C until analysis.

Patients were divided into two groups based on the presence or absence of clinical and laboratory signs of cholestasis at the moment of inclusion: Group 1 - infants with clinical and laboratory symptoms of cholestasis by C-triol and ChT analysis and; Group 2 - infants without clinical or laboratory symptoms of cholestasis/cytolysis syndrome at the time of oxysterol measurement, but who had transient cholestasis during their first months of life.

Group 1 included 80 children (mean age 3.0 months, SD 1.7 months, range 0.5–10 months). 65 children (81%) of Group 1 had an enlarged liver at the time of oxysterol and ChT measurement (mean 4.3 cm, SD 1.6 cm, range 2.5–8.0 cm) below the costal level along the mid-clavicular line, and 71 (89%) had splenomegaly (mean 2.5 cm, SD 1.6 cm, range 0.5–6.0 cm). Increased biochemical markers of cholestasis and cytolysis syndrome were observed for all children in Group 1 (Table 1).

**Table 1:** Markers of liver function at the time of C-triol and ChT measurement

Indicators	Group 1 (*n* = 80)	Group 2 (*n* = 28)
	Mean (Q1;Q3)	
ALT, U/l (Normal range: 0–40)	150 (98–245)	34 (16–42)
AST, U/l (Normal range: 0–40)	228 (128–322)	22 (12–46)
Total bilirubin, μM/l (Normal range: 3.4–21.0)	157 (105–203)	13.2 (4.1–16.4)
Direct bilirubin, μM/l (Normal range: 0–5.5)	81 (51–111)	4.3 (3.1–5.0)
GGT, U/l (Normal range: 0–50)	194 (93–398)	21 (12–33)
ALP, U/l (Normal range: 50–360)	535 (427–720)	219 (112–280)
Cholesterol, mM/l (Normal range: 3.1–5.2)	5.7 (3.7–7.3)	3.2 (2.1–4.3)
Triglycerides, mM/l (Normal range: 0.6–1.7)	1.4 (0.9–2.0)	1.1 (0.6–1.3)

*ALT* alanine aminotransferase, *ALP* alkaline phosphatase, *AST* aspartate aminotransferase, *GGT* gamma-glutamyltransferase, Q1:Q3, first and third quartile values

Group 2 included 28 children (mean age 31.8 months, SD 28.0 months, range 5–84 months) with details of previous neonatal cholestasis from medical histories. All children from Group 2 had a history of jaundice and hepatomegaly during the first months of life. Twenty patients had a history of previous splenomegaly and episodes of acholic stool. Jaundice termination mean age 3.7 months, SD 1.8 months (with simultaneous normalization of stool colour, bilirubin levels, GGT activity and cholesterol levels). No cholestasis detected at the examination time, but seven children reveals hepatosplenomegaly.

No signs of hepatic insufficiency and portal hypertension detected in Group 1 or 2 during the observation period.

A clinical description of the confirmed NP-C case: the child (XY) was born at 39 weeks. At birth: weight 3670 g, height 50 cm, Apgar 7/8, liver + 2–2.5 cm, spleen 2 cm. Day 2: jaundice appeared. Day 4: liver + 4–4.5 cm, spleen 3.5–4 cm, tot.bil 124 mkM/L, dir.bil 33 mkM/L, Alp 700 mkM/L, ALТ 173 U/L, AST 118 U/L, TORCH negative, no signs of portal hypertension. Jaundice resolved by 4 weeks, liver reduced by 6 months (but the spleen gradually increased to 5–5.5 cm). Muscular hypotension was noted at 8 weeks. 12 months: some delay in psycho-motor development. At the age of 2.5 years (when the patient had initial neurological signs of the disease) Miglustat therapy was started with positive effect. Family anamnesis without features (sibs – healthy girl 7 years old).

### Clinical examination and general laboratory tests

Examination included: presence of jaundice, stool colour, pruritus, spleen size and liver size (cm below the right costal level and along the right mid-clavicular line). Laboratory tests were: total and direct bilirubin levels, gamma glutamyl transpeptidase (GGT) and alkaline phosphatase (ALP) enzyme activities, serum cholesterol and triglycerides, raised transaminases (alanine aminotransferase [ALT] and aspartate aminotransferase [AST] activities) and liver function tests (albumin, cholinesterase [CE], fibrinogen, prothrombin time and INR).

### C-triol analysis

EDTA-plasma C-triol was determined for all patients as dimethylaminobutyrate esters by liquid chromatography tandem mass spectrometry (LC-MS/MS) analysis, with small modifications [25]. Chromatographic separation was performed on a Phenomenex Gold C18 column (2.1 × 100 mm, 5 μm) using a linear gradient of 5 mM ammonium formate and acetonitrile on an LC20 HPLC system (Shimadzu, Japan). This was followed by detection on a Sciex 3200 QTrap mass spectrometer (ABSciex, USA). D7-C-triol was used as an internal standard. The assay was linear over a concentration range of 0.5–200 ng/mL. Intra- and inter-day assay variation varied between 2.3–9.6% and 3.8–11.8% (% CV) respectively. Limits of quantification (LOQ) and detection (LOD) were 0.5 ng/mL and 0.05 ng/mL, respectively. Normal C-triol values were 0–50 ng/mL [18, 26].

### Lysosomal enzymes activity

#### Chitotriosidase assay

ChT activity was measured based on dried blood spots prepared on filter cards from EDTA samples. A standard fluorometric method was used, as described [27]. The normal range of ChT values was 2.5–100 nM/h/ml.

#### Beta-glucosidase and sphingomyelinase activities

Beta-glucosidase and sphingomyelinase activities were determined as a part of a multiplex analysis according to published protocol with some modifications [28]. Mass spectrometry was performed on an API 3200 QTrap tandem mass spectrometer (ABSciex, USA) in multiple-reaction monitoring (MRM) mode. Measurements were standardized against control samples with known enzyme activities obtained from the Center for Disease Control (CDC; Atlanta, USA).

### Molecular genetic analysis

Mutation analysis was performed using DNA from patients’ blood samples. Exons and flanking regions of *NPC1* and *NPC2* genes were PCR amplified using an original PCR primers (Table 2) and sequenced at the ABI PRISM 3500xL genetic analyser (Applied Biosystems, USA) with a standard BigDye chemistry.

**Table 2:** Primers for PCR amplification and Sanger-sequencing of exons and flanking regions of NPC1 and NPC2 genes

Exon #	Primer	PCR fragment size	Primers Tm
1	NPC1F 5′-GAGCCAGACTCCATAAGTC-3′ NPC1R 5′-AGACCAACTTCCCCAGGAC-3′	466	64
2	NPC2F 5′-GATTGTACTTGAGTGGGCAC-3′ NPC2R 5′-ACAGAGGATCTTGTGATCAG-3′	238	62
3	NPC3F 5′-TGAGGAATGTTGACCTTACTCTAAC-3′ NPC3R 5′-GAAAGCTGAGCATTACCAGTTC-3’	207	64
4	NPC4F 5’-GCTGGCCCTATTATGTGTGAG-3′ NPC4R 5′-ATTTCCTGGCCAATGGAACTG-3’	312	64
5–6	NPC5F 5’-CAGCATTCCAGCATGGTGCATATG-3′ NPC6R 5′-CCATGCAATGGTATTCATGGAGG-3’	1126	64
7–8	NPC7F 5’-GAAGGCAGTAATTAGGGAGG-3′ NPC8R 5′-CCACAAGGTCATCTAGAGTG-3’	1108	62
9	NPC9F 5’-GCTGATTAATCAAGATCTGAGAG-3′ NPC9R 5′-CTCACCTCTGGGTTATGCTC-3’	367	64
10	NPC10F 5’- GCTGAGCTGTATTACTCAACTG-3′ NPC10R 5′-TACCACTTGATGCTAATGAC-3’	292	64
11	NPC11F 5’- CAGAGATACAGTCCATAGCTC-3′ NPC11R 5′- GAGCTGAGATTCAGTCACTG-3’	501	62
12–13	NPC12F 5’- CTTTGTATCGTGAAAGTTAG-3′ NPC13R 5′-CCAGGAGCCATTCACAGTC-3’	931	60
14	NPC14F 5’- CTGCTGTAGAAGGTGGTCTC-3′ NPC14R 5′- GACATGTTCAGGTAGCCAGC-3’	507	64
15–16	NPC15F 5’-CTTGTATCTGTACATGCACATG-3′ NPC16R 5′-GATAATCTGTTTCAGTGAGAGG-3’	472	64
17	NPC17F 5’-GCCCTGTACTCCCTATTAGC-3′ NPC17R 5′-GTTAGAAGCAGGCACTTGCTT-3’	298	62
18–19	NPC18F 5’-GAATCATGAGTCCAGCTGGAG-3′ NPC19R 5′-GGGAGACCCAGCTTTGATATAC-3’	876	62
20	NPC20F 5’- GAAAGTGACATGTGGCTGAAG-3′ NPC20R 5′- GTGGATGCTTATCTGCAATGGC-3’	315	60
21	NPC21F 5’- CAAGACCTGGACTCTCTTGAC-3′ NPC21R 5′- GATATACTGCCCTGTGCTCAG-3’	357	62
22	NPC22F 5’- AGGAGTCTGACCACTTGGCAGT-3′ NPC22R 5′- ACATGGAATCTAAGACAGCC-3’	382	64
23	NPC23F 5’-GAGGCCTTGTAAGTCCAATGGG-3′ NPC23R 5′-GTACAGGATCCAGACTCTTCAG-3’	312	64
24	NPC24F 5’- GAGAAATCCTTGTAAGGAAG-3′ NPC24R 5′- GATGAGAACTCTTACCTATG-3’	228	64
25	NPC25F 5’-TTCCAAAGTGGGATTACAGGCGTG-3′ NPC25R 5′-GACCGACCCTTAGACACAGTTCAG-3’	183	64
1	NC2-1F 5’-AGACTGCAGGCTTCTGGGCCTGAG-3′ NC2-1R 5′-CCAGCCCCAGGGGTCTCAGCGC-3’	332	64
2	NC2-2F 5’-AGCAGAGCACCTTCCCATTAG G-3′ NC2-2R 5′-CTCCCCTCCATTCCCATGCTT A-3’	256	64
3	NC2-3F 5’-ATGCTGTTGCTTGGGATTATTTC-3′ NC2-3R 5′-CCCATCTCTGCTTCTTGCCCACT-3’	339	62
4	NC2-4F 5’-GGCTGTAAGCTGTGCCCACATGCT-3′ NC2-4R 5′-CTGGACCTTCCTTACTCCGACAG-3’	552	62
5	NC2-5F 5’-TAACTTGCCCTAGGGTTATTGC- 3′ NC2-5R 5′-GTGCACTCTGGGACCACGGAACT-3’	505	62

Exons and flanking regions of genes included in the customized Ion AmpliSeq DNA panel (*ABCB11, ABCB4, ABCD3, AGL, AKR1D1, ALAD, ALDOB, ATP7B, ATP8B1, C10orf2, CYP7B1, DGUOK, FAH, FBP1, G6PC, GAA, GALE, GALT, GBE1, GYS2, HADHA, JAG1, LIPA, MPI, MPV17, OTC, PFKM, PGAM2, PGM1, PHKA2, PHKB, PHKG2, POLG1, PYGL, SERPINA1, SLC25A13, SLC37A4, TALDO1, TJP2, BCS1L, NBAS, SERAC1, TRMU, SCO1, LARS, SMPD1,* and *GBA*) were amplified by Ion AmpliSeq™ Library Kit 2.0 and sequenced at the Ion Torrent PGM™ System (Thermo Fisher Scientific, USA). Alignment to the reference genome and the search for differences was performed using the server Torrent Suite, plugin Variant Caller.

### Data analysis

Statistica (version 10, StatSoft Inc., USA) and RStudio Desktop (version 1.2.1335, RStudio, USA) were used for statistical analysis. Results are presented using descriptive statistics (arithmetic means ± standard deviations, ranges (minimum–maximum), and quartiles where appropriate). Pearson and Spearman correlation methods were used for plasma C-triol and cholesterol correlation. Statistical significance was assumed for r values at the two-tailed 5% level.

## Results

**Fig. 1:**
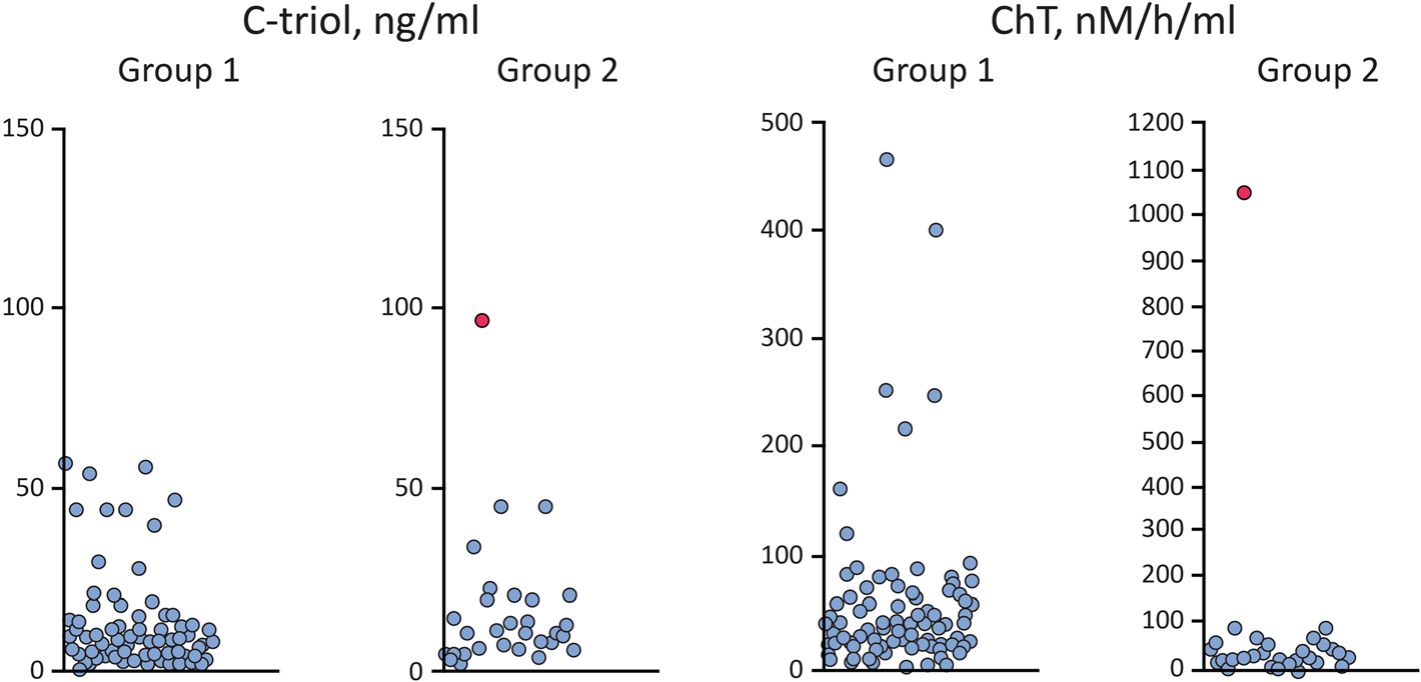
Distribution of C-triol concentration and ChT activity. Footnote: The red-shaded data point signifies the NP-C patient

As observed in Table 3, C-triol was elevated in three patients and ChT – in seven patients from Group 1 (one patient demonstrates both C-triol and ChT) (Fig. 1). For Group 2 increased C-triol and ChT were observed in one patient who was subsequently diagnosed with NP-C by molecular genetic testing.

**Table 3:** Mutations and diagnoses among children with elevated plasma C-triol and/or ChT

Patient No.	C-triol	ChT	Mutations identified	Main diagnosis
Group 1				
1	57.8	0	*JAG1*: c.2384delG (p.G795 fs) ClinVar SUB5165272	Alagille syndrome
2	56.6	224	*LARS*: rs34823161 (c.3077A > G, Tyr1026Cys)	Clinical significance not clear (could not be a cause of an autosomal recessive disorder)
3	55.0	27	*ABCB11*: rs72549402 (c.1445A > G, Asp482Gly) and rs1459273753 (c.2178 + 1G > A)	Byler disease, compound heterozygosity confirmed
4	9.9	126	Not found	Cholestatic liver disease, NOS aetiology
5	5.8	166	Not found	Cholestatic liver disease, NOS aetiology
6	48.0	407	Not found	Biliary atresia
7	16.6	253	Not found	Biliary atresia
8	9.8	258	Not found	Biliary atresia
9	7.6	469	Not found	Biliary atresia
Group 2				
10	97.6	1056	*NPC1*: rs886053665 (c.2090 T > C, Val697Ala) and rs786200877 (c.3591 + 1G > A)	NP-C, compound heterozygosity confirmed

*NOS* not otherwise specified. Normal central laboratory values for plasma C-triol 0–50 ng/ml and ChT 2.5–100 nM/h/ml

All 9 patients with elevated plasma C-triol and/or ChT from Group 1 and one from Group 2 were analysed for mutations in *NPC1*, *NPC2* and 47 additional liver damage panel genes (see above). For 4 (of 10) patients clinically relevant mutations were detected, including new heterozygous *JAG1* mutation of presumably Armenian origin (Table 3). For patients #3 and #10 the compound heterozygosity was confirmed by ancestors sequencing.

**Fig. 2:**
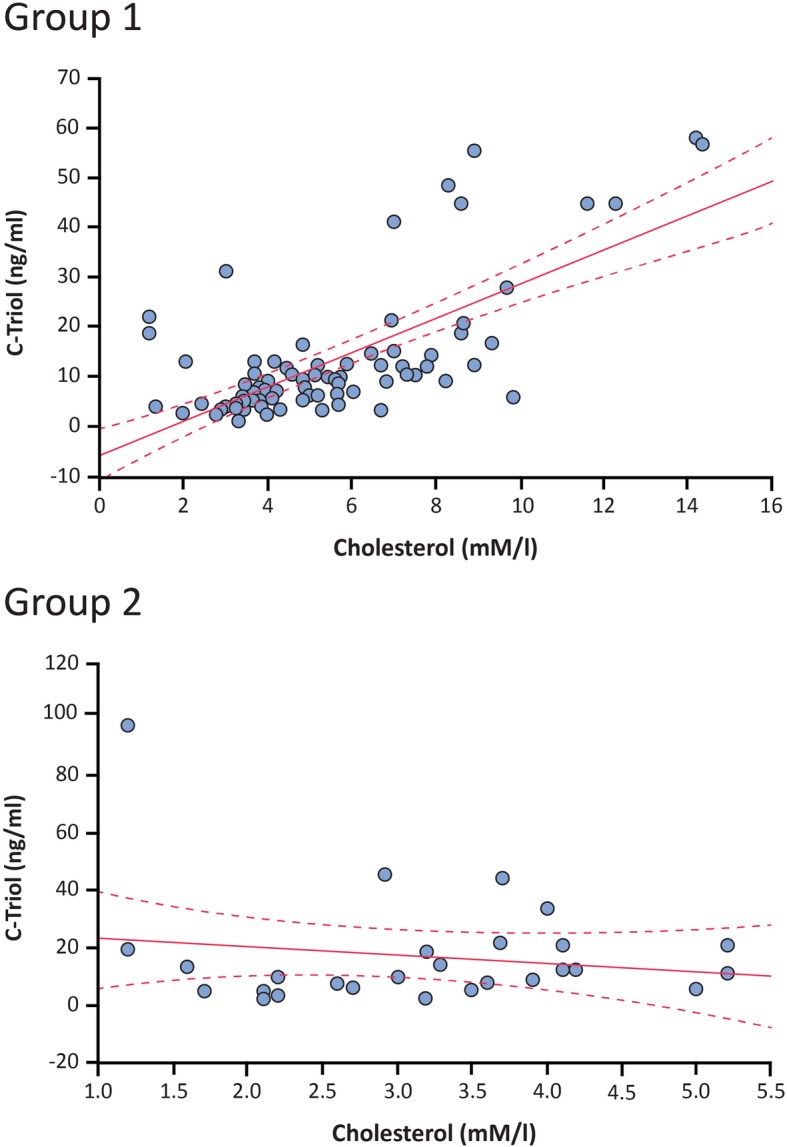
Correlation analysis between C-triol and blood cholesterol levels. Footnote: The solid red lines are the least-squares regression lines; the dotted red lines represent the 95% confidence interval

All patients with elevated levels of ChT demonstrate normal range of lysosomal acid lipase, beta-glucosidase, and sphingomyelinase in dried blood spots (data not shown). The linear regression analysis showed a statistically significant positive correlation between cholesterol and C-triol levels in Group 1 (*r* = 0.69, *p* < 0.05) and no correlation in Group 2 (*r* = − 0.17, *p* > 0.05) (Fig. 2). The correlation between cholesterol and C-triol levels in Group 1 was confirmed using a non-parametric Spearman correlation test (*ρ* = 0.586, *p* < 0.05).

## Discussion

C-triol is an oxidative derivative of cholesterol that can potentially be elevated due to hypercholesterolemia of various aetiologies (including cholestasis syndrome).

In a previous prospective cohort study, Polo et al. assessed the specificity of C-triol and 7-KC in neonates with severe cholestasis and suspected NP-C [23]. Levels of both oxysterol markers were significantly higher in 6/7 patients compared with healthy controls, but a genetic diagnosis of NP-C was only confirmed in 1/6 patient. Biliary atresia was diagnosed in the other five patients with high-oxysterol levels, and one patient with the lowest measured oxysterol levels had transient neonatal cholestasis. There were no correlations between oxysterol levels and direct bilirubin among test subjects. A further control group of adults with cholestatic liver diseases showed high oxysterol levels in 5/15 cases, and levels significantly exceeded the normal range in two cases. It was concluded that among the neonates with cholestasis where NP-С was not confirmed, increased oxysterol levels were related to oxidative stress associated with cholestasis but were not specific for NP-C. This hypothesis was in line with earlier studies where oxysterol levels were measured in adults with hepatitis C virus infection [29, 30].

In this study the neonates and infants from Group 1 demonstrates strong correlation between C-triol and cholesterol levels. False positive results from either C-triol or ChT measurements were detected in 9 (11.3%) patients with cholestasis syndrome at the time of measurement (Group 1). In each case, such findings were related to congenital and/or hereditary liver diseases associated with cholestasis of varying severity. An increase in C-triol and ChT was detected in only one child in Group 2 in whom the cholesterol level was normal, and who was subsequently diagnosed with NP-C. Other congenital diseases were confirmed in a number of other patients with elevated levels of these biomarkers. Thus combined evaluation of plasma oxysterols and ChT (possibly together with the other biomarkers, such as lysosphingolipids and specific bile acids [31]), particularly in cases where cholesterol is not elevated, may serve as a useful screening approach for identifying new NP-C cases, and can help detect candidates for molecular genetic testing.

## Conclusion

As with a number of inherited neurodegenerative diseases, the early identification of new cases is particularly important in NP-C as targeted therapies now exist that are capable of slowing neurological deterioration. Combined analysis of ChT activity and C-triol levels may be an effective method to identify NP-C, but the positive predictive value in the context of neonatal cholestasis is low. The major limitation of our study is that only one patient diagnosed with NP-C (in Group 2, patients with previous neonatal cholestasis).

## Data Availability

The datasets used and/or analysed during the current study are available from the corresponding author on reasonable request. Also some data supporting our findings can be found at: http://www.med-gen.ru/docs/diss_Proshlyakova_TY.pdf

## Abbreviations

7-KC: 7-ketocholesterol
ChT: Chitotriosidase
C-triol: Cholestane-3β,5α,6β-triol
GD: Gaucher disease
LC-MS/MS: Liquid chromatography-tandem mass spectrometry
LSDs: Lysosomal storage diseases
NGS: Next-generation sequencing
NP-C: Niemann-Pick disease type C
ROS: Reactive oxidative species
SD: Standard deviation
